# Caregiving in Older Adulthood and Meaning in Life

**DOI:** 10.1093/geroni/igab046.2978

**Published:** 2021-12-17

**Authors:** Zainab Suntai

**Affiliations:** University of Alabama, Tuscaloosa, Alabama, United States

## Abstract

While most of the literature on caregiving in adulthood focuses on grandparenting, there is an increasing trend of older adults providing care to an adult care recipient. Older caregivers are often females who are providing care to a spouse with medical conditions and are often doing so while coping with their own functional limitations, with limited support. Within the older adult literature, studies have captured the strain experienced by caregivers of older adults, who are often burdened by the loss of time and opportunity as a result of caregiving. For caregivers in older adulthood however, caregiving may be an avenue to remain engaged and active, and a way to have purpose in life. Therefore, the purpose of this study was to examine the association between caregiving in older adulthood and meaning in life. Data from the 2018 National Health and Aging Trends study were used, which is an annual longitudinal panel survey of Medicare beneficiaries in the United States. Chi square tests were used for bivariate analyses and a logistic regression model was used to predict meaning in life based on caregiving status. After accounting for all other explanatory variables, caregivers were 50% more likely to have meaning in life compared to non-caregivers (OR=1.501, CI= 1.493-1.510). This indicates that caregiving can be a form of meaningful/productive engagement for older adults and interventions should focus on providing adequate emotional and financial support to older caregivers.

